# 2337. Immunogenicity and Safety of a Heterologous Booster Dose of Omicron Subvariant (BA.1 and BA.5) and Bivalent SARS-CoV-2 Recombinant Spike Protein Vaccines: A Phase 3, Randomized, Clinical Trial

**DOI:** 10.1093/ofid/ofad500.1959

**Published:** 2023-11-27

**Authors:** Chijioke Bennett, Wayne Woo, Alex Marcheschi, Raburn M Mallory

**Affiliations:** Novavax, Gaithersburg, Maryland; Novavax, Inc., Gaithersburg, Maryland; Novavax, Inc, Durham, North Carolina; Novavax, Gaithersburg, Maryland

## Abstract

**Background:**

Currently circulating Omicron subvariants contain mutations permitting evasion of neutralization with prototype vaccines. Novel Omicron BA.1 (NVX-CoV2515) and Omicron BA.5 (NVX-CoV2540) subvariant-specific vaccines were tested alone or as bivalent preparations combined with the prototype vaccine (NVX-CoV2373) to determine if superior antibody responses to variants of concern are achieved.

**Methods:**

Healthy adults previously immunized with 3 doses of prototype mRNA vaccines (BNT162b2 or mRNA-1273) were enrolled in a phase 3 study (NCT05372588) to investigate heterologous boosting with adjuvanted SARS-CoV-2 recombinant spike protein vaccines. In Part 1, these participants were randomized 1:1:1 to receive a single dose of NVX-CoV2373, NVX-CoV2515, or a bivalent mixture. In Part 2, participants were randomized 1:1:1 to receive a single dose of NVX-CoV2373, NVX-CoV2540, or a bivalent mixture. Immunogenicity was assessed 14 and 28 days after vaccine administration for the SARS-CoV-2 ancestral strain, Omicron BA.1 and BA.5 sublineages, and relative safety of each vaccine construct was assessed.

**Results:**

In Part 1, 829 participants received trial vaccines; the monovalent NVX-CoV2515 vaccine demonstrated a superior Day 14 neutralizing antibody response to BA.1 compared to NVX-CoV2373 (MN_50_ titers [95% CI] of 130.8 [109.2, 156.7] and 83.9 [69.6, 101.2], respectively) (**Fig 1**). Anti-spike IgG antibody responses to BA.1 were similar across all groups with antibody levels of 30,170.9 (25,663.7, 35,469.6), 24,174.8 (20,943.6, 27,904.6), and 23,045.5 (20,113.5, 26,404.8) EU/mL for NVX-CoV2373, NVX-CoV2515, and the bivalent vaccine, respectively (**Fig 2**). All three formulations were similarly well-tolerated. Part 2 is ongoing.

Figure 1. Immunogenicity against ancestral and BA.1 variant strains of SARS-CoV-2, following booster vaccination with NVX-CoV2515, NVX CoV2373, or Bivalent NVX-CoV2373 + NVX-CoV2515.

**Figure:**
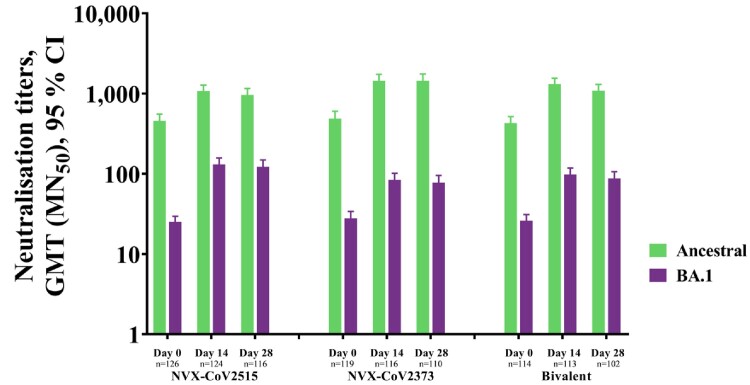

Microneutralization titers for the ancestral and BA.1 variant.

Figure 2. Immunogenicity against ancestral and BA.1 variant strains of SARS-CoV-2, following booster vaccination with NVX-CoV2515, NVX CoV2373, or Bivalent NVX-CoV2373 + NVX-CoV2515.

**Figure:**
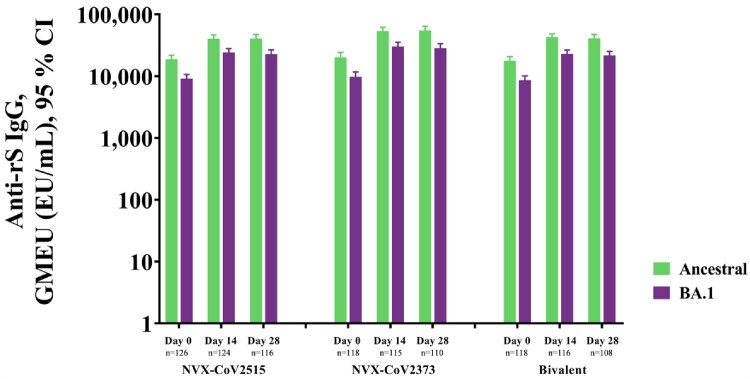

Anti-rS IgG titers for the ancestral and BA.1 variant.

**Conclusion:**

The study met its primary endpoint with the monovalent BA.1 vaccine, NVX-CVoV2515, demonstrating superior neutralizing antibody responses for the BA.1 subvariant. NVX-CoV2373 induced robust immune responses to ancestral and Omicron subvariant strains of SARS-CoV-2 when administered as a heterologous booster dose. Overall, immunogenic responses were similar to those for the NVXCoV2515 and the bivalent vaccines. Safety data based on solicited and unsolicited AEs were consistent with the established safety profile of NVX-CoV2373.

**Disclosures:**

**Chijioke Bennett, MD, MPH, MBA**, Novavax, Inc.: employee|Novavax, Inc.: Stocks/Bonds **Wayne Woo, MS**, Novavax, Inc.: employee|Novavax, Inc.: Stocks/Bonds **Alex Marcheschi, PharmD**, Novavax, Inc: Employee|Novavax, Inc: Stocks/Bonds **Raburn M. Mallory, M.D.**, Novavax: Stocks/Bonds

